# Supplementary material for: Comparative diagnostic accuracy between simplified and original flow cytometric gating strategies for peripheral blood neutrophil myeloperoxidase expression in ruling out myelodysplastic syndromes
Source: PLoS One. 2022 Nov 18;17(11):e0276095. doi: 10.1371/journal.pone.0276095 (PMC9674135; doi:10.1371/journal.pone.0276095)
Supplement: S1 Table — (DOCX) [file pone.0276095.s001.docx]

**Table S1. Technical information for reagents used for immunocytochemical staining.**

| Marker | Fluorochrome | Clone | Source | Catalogue number | (volume per test) |
| --- | --- | --- | --- | --- | --- |
| CD15 | PerCP-Cy5.5 | HI98 | BD Biosciences | 560828 | 2.5 μL |
| CD11b | APC | D12 | BD Biosciences | 3331443 | 2.5 μL |
| CD16 | APC-H7 | 3G8 | BD Biosciences | 560195 | 2.5 μL |
| CD14 | V450 | MФP9 | BD Biosciences | 560349 | 2.5 μL |
| CD45 | V500 | HI30 | BD Biosciences | 560777 | 2.5 μL |
| MPO | PE | 5B8 | BD Biosciences | 333139 | 10 μL |
| BD IntraSure^TM^ Kit | - | - | BD Biosciences | 641778 | 100 μL of Reagent A  50 μL of Reagent B |
| BD FACS^TM^ lysing solution | - | - | BD Biosciences | 349202 | 2 mL 1X |
