# Supplementary material for: Comparative diagnostic accuracy between simplified and original flow cytometric gating strategies for peripheral blood neutrophil myeloperoxidase expression in ruling out myelodysplastic syndromes
Source: PLoS One. 2022 Nov 18;17(11):e0276095. doi: 10.1371/journal.pone.0276095 (PMC9674135; doi:10.1371/journal.pone.0276095)
Supplement: S2 Table — (DOCX) [file pone.0276095.s002.docx]

**Table S2. Overview of lasers for FACSCanto-II ^TM^ flow cytometer.***

| Laser | Emission wavelength, nm | Power, mW |
| --- | --- | --- |
| Violet | 405 | 30 |
| Blue | 488 | 20 |
| Red | 633 | 17 |

* Dynamic range, 18-bit (262,144 channels)
