# Supplementary material for: Comparative diagnostic accuracy between simplified and original flow cytometric gating strategies for peripheral blood neutrophil myeloperoxidase expression in ruling out myelodysplastic syndromes
Source: PLoS One. 2022 Nov 18;17(11):e0276095. doi: 10.1371/journal.pone.0276095 (PMC9674135; doi:10.1371/journal.pone.0276095)
Supplement: S3 Table — (DOCX) [file pone.0276095.s003.docx]

**Table S3. Agreement of binary intra-individual robust coefficient of variation for peripheral blood neutrophil myeloperoxidase expression between simplified and original flow cytometric gating strategies after excluding two chronic myelomonocytic leukemia cases from the study sample (Cohen’s Kappa coefficient = 1.00).**

| Original gating strategy | Simplified gating strategy | |  |
| --- | --- | --- | --- |
|  | RCV < 30% | RCV ≥ 30% | Total |
| RCV < 30% | 24 | 0 | 24 |
| RCV ≥ 30% | 0 | 36 | 36 |
| Total | 24 | 36 | 60 |

Abbreviations: RCV = robust coefficient of variation.
