# Supplementary material for: Comparative diagnostic accuracy between simplified and original flow cytometric gating strategies for peripheral blood neutrophil myeloperoxidase expression in ruling out myelodysplastic syndromes
Source: PLoS One. 2022 Nov 18;17(11):e0276095. doi: 10.1371/journal.pone.0276095 (PMC9674135; doi:10.1371/journal.pone.0276095)
Supplement: S4 Table — (DOCX) [file pone.0276095.s004.docx]

**Table S4. Comparative diagnostic accuracy of intra-individual robust coefficient of variation for peripheral blood neutrophil myeloperoxidase expression between simplified and original flow cytometric gating strategies after excluding two chronic myelomonocytic leukemia cases from the study sample (n = 53).**^a^

|  | Flow cytometric gating strategy | | | | | | | |
| --- | --- | --- | --- | --- | --- | --- | --- | --- |
|  | Original | | | | Simplified | | | |
| Intra-individual RCV, %, median (range) |  | |  | |  | |  | |
| Confirmed suspicion of MDS (n = 21) | 37.4 | (30.7–54.1) | | 37.5 | | (30.8–54.4) | |  |
| Unconfirmed suspicion of MDS (n = 32) | 29.2 | (24.7–37.8) | | 29.2 | | (24.7–37.9) | |  |
| Area under ROC curve (95% CI) | 0.92 | | (0.82–0.98) | | 0.92 | | (0.82–0.98) | |
| RCV ≥ 30% |  | |  | |  | |  | |
| True positive, *n* | 21 | | … | | 21 | | … | |
| False positive, *n* | 13 | | … | | 13 | | … | |
| False negative, *n* | 0 | | … | | 0 | | … | |
| True negative, *n* | 19 | | … | | 19 | | … | |
| Sensitivity, % (95%CI) | 100 | | (84–100) | | 100 | | (84–100) | |
| Specificity, % (95%CI) | 59 | | (41–76) | | 59 | | (41–76) | |
| PPV, % (95%CI) | 62 | | (44–78) | | 62 | | (44–78) | |
| NPV, % (95%CI) | 100 | | (82–100) | | 100 | | (82–100) | |

Abbreviations: CI = confidence interval; MDS = myelodysplastic syndrome; NPV = negative predictive value; PPV = positive predictive value; RCV = robust coefficient of variation; ROC = receiver operating characteristics.

^a^ The analytical sample consisted of 21 and 32 patients with confirmed and unconfirmed suspicions of myelodysplastic syndrome, after excluding two chronic myelomonocytic leukemia cases and seven patients with uninterpretable bone marrow cytomorphology at baseline.
