# Supplementary material for: Comparative diagnostic accuracy between simplified and original flow cytometric gating strategies for peripheral blood neutrophil myeloperoxidase expression in ruling out myelodysplastic syndromes
Source: PLoS One. 2022 Nov 18;17(11):e0276095. doi: 10.1371/journal.pone.0276095 (PMC9674135; doi:10.1371/journal.pone.0276095)
Supplement: S5 Table — (DOCX) [file pone.0276095.s005.docx]

**Table S5. Agreement of binary intra-individual robust coefficient of variation for peripheral blood neutrophil myeloperoxidase expression between simplified and original flow cytometric gating strategies in external validation sample comprising patients with milder level of peripheral blood cytopenia (Cohen’s Kappa coefficient = 1.00).**

| Original gating strategy | Simplified gating strategy | |  |
| --- | --- | --- | --- |
|  | RCV < 30% | RCV ≥ 30% | Total |
| RCV < 30% | 11 | 0 | 11 |
| RCV ≥ 30% | 0 | 15 | 15 |
| Total | 11 | 15 | 26 |

Abbreviations: RCV = robust coefficient of variation.
