# Supplementary material for: Comparative diagnostic accuracy between simplified and original flow cytometric gating strategies for peripheral blood neutrophil myeloperoxidase expression in ruling out myelodysplastic syndromes
Source: PLoS One. 2022 Nov 18;17(11):e0276095. doi: 10.1371/journal.pone.0276095 (PMC9674135; doi:10.1371/journal.pone.0276095)
Supplement: S6 Table — (DOCX) [file pone.0276095.s006.docx]

**Table S6. Comparative diagnostic accuracy of intra-individual robust coefficient of variation for peripheral blood neutrophil myeloperoxidase expression between simplified and original flow cytometric gating strategies in external validation sample comprising patients with milder level of peripheral blood cytopenia (n = 23).**^a^

|  | Flow cytometric gating strategy | | | | | | | |
| --- | --- | --- | --- | --- | --- | --- | --- | --- |
|  | Original | | | | Simplified | | | |
| Intra-individual RCV, %, median (range) |  | |  | |  | |  | |
| Confirmed suspicion of MDS (n = 4) | 32.4 | (30.2–34.9) | | 32.5 | | (30.2–35.1) | |  |
| Unconfirmed suspicion of MDS (n = 19) | 29.8 | (25.8–37.5) | | 29.8 | | (25.8–37.9) | |  |
| Area under ROC curve (95% CI) | 0.82 | | (0.61–0.95) | | 0.82 | | (0.61–0.95) | |
| RCV ≥ 30% |  | |  | |  | |  | |
| True positive, *n* | 4 | | … | | 4 | | … | |
| False positive, *n* | 8 | | … | | 8 | | … | |
| False negative, *n* | 0 | | … | | 0 | | … | |
| True negative, *n* | 11 | | … | | 11 | | … | |
| Sensitivity, % (95%CI) | 100 | | (40–100) | | 100 | | (40–100) | |
| Specificity, % (95%CI) | 58 | | (34–80) | | 58 | | (34–80) | |
| PPV, % (95%CI) | 33 | | (10–65) | | 33 | | (10–65) | |
| NPV, % (95%CI) | 100 | | (72–100) | | 100 | | (72–100) | |

Abbreviations: CI = confidence interval; MDS = myelodysplastic syndrome; NPV = negative predictive value; PPV = positive predictive value; RCV = robust coefficient of variation; ROC = receiver operating characteristics.

^a^ The external validation sample consisted of four and 19 patients with confirmed and unconfirmed suspicions of myelodysplastic syndrome, after excluding three patients with uninterpretable bone marrow cytomorphology at baseline.
