# Supplementary material for: Comparative diagnostic accuracy between simplified and original flow cytometric gating strategies for peripheral blood neutrophil myeloperoxidase expression in ruling out myelodysplastic syndromes
Source: PLoS One. 2022 Nov 18;17(11):e0276095. doi: 10.1371/journal.pone.0276095 (PMC9674135; doi:10.1371/journal.pone.0276095)
Supplement: Attachment — Submitted filename: Raskovalova_Response_to_Reviewers_220905.doc [file pone.0276095.s008.doc]

**Reviewer #1**

1. **Figure 1G: Following up on my last comment about the gating threshold in this plot, but now more specifically. What are the gray dots in this plot (I noticed these when I downloaded the Figure and looked very closely)? Are those MPO negative granulocytes and shouldn’t those be included in your analysis as well, as degranulation occurred in these granulocytes? Excluding granulocytes with low MPO expression levels creates a bias in your RCVs (although small in the displayed case). However, if there are more granulocytes with such low MPO expression levels, readers will exclude those based on the method displayed here, which is incorrect and will tremendously influence the resulting RCVs. To address this comment I would like to see the following: Please analyze the RCVs on all granulocytes without excluding any based on MPO expression levels. This will prevent any potential bias in the RCVs of MPO expression in the granulocytes.**

We thank this Reviewer for the opportunity to clarify this point. Actually, RCV is robust to outliers and constitutes an alternative to the coefficient of variation for skewed distributions (See Bonett and Seier. *Biomed J* 2005 and Arachchige et al. *J Appl Stat* 2020). To address this Reviewer’s concern, we computed RCV based on all granulocytes (Panel A) and after excluding granulocytes with lower levels of MPO expression (Panel B), respectively. Reassuringly, the RCV value was 27.6 for both experiments.

| Panel A. All granulocytes | Panel B. Excluding granulocytes with lower level of MPO expression |
| --- | --- |
|  |  |
| RCV = 27.6 | RCV = 27.6 |

We agree with this Reviewer that the original gating strategy is somewhat subjective and operators with varying levels of expertise might introduce bias in estimating RCV values. In contrast, the gated cells are selected based on CD16 / CD11b double positivity, using the simplified gating strategy (Panel C).

Panel C. Simplified gating strategy

RCV = 27.6

Changes in the revised manuscript in response to the above are highlighted below:

Simplified gating strategy (page 9):

“Mature neutrophils were individualized (CD16+ CD11b+) and gated cells were selected based on CD16/CD11b double positivity (D). The MPO SSC dot plot (E) was used only to visualize MPO expression of mature neutrophils.”

Myeloperoxidase expression (page 9):

“RCV is robust to outliers and constitutes an alternative to the coefficient of variation for skewed distributions.”

Discussion (page 20):

“Third, the original gating strategy was somewhat subjective and operators with varying levels of expertise might introduce bias in estimating RCV values. Reassuringly, the RCV value was rather unchanged after excluding granulocytes with lower levels of MPO expression.”

**Minor**

2. **P5 Line 61: Please leave out Indeed in the beginning of the sentence.**

This term has been removed from the revised manuscript.

3. **P5 Line 68: This part of the sentence is not correctly written: “that constitutes the major component of neutrophil azurophilic granules”.**

This sentence has been rephrased.

4. **P5 Line 70: “MPO cytoplasmic expression” should be corrected to “”cytoplasmic expression of MPO”**

This has been revised accordingly.

5. **P5 Line 71: “immuno-cytochemical” should be corrected to “immunocytochemical”**

This has been corrected.

6. **P5 Line 76: Thank you for adding this passage to the manuscript. One small remark about part of this sentence: “relied on operator’s expertise”. Perhaps the authors can correct this to: “relied on the expertise of the operators”.**

We have edited this sentence accordingly.

7. **P6 Line 104: In this part of the sentence “we assessed agreement and comparative accuracy of RCV”, the abbreviation of RCV is first used and should be written in full with abbreviation between brackets. I also miss a word in the sentence. Please correct this part of the sentence to: “we assessed the agreement and comparative accuracy of the RCV.”**

Actually, the first occurrence of RCV is located Page 5, line 87. The sentence has been corrected accordingly.

8. **P7 Line 199-128: Please remove the bullet points and write this down in narrative form.**

We have removed the bullet points from this para.

9. **P10 Line 219-222: Please remove the comma after “flow cytometric scoring”. That comma is not necessary.**

We have removed the comma from this sentence.

10. **Figure 2: Please add a plot displaying the MPO expression in granulocytes here too for completeness.**

We have modified Figure 2, as requested by this Reviewer.
